# Nuclear Magnetic Resonance‐based fragment screen of the E3 ligase Fem‐1 homolog B

**DOI:** 10.1002/pro.70365

**Published:** 2025-11-13

**Authors:** Jade M. Katinas, Kangsa Amporndanai, Ashley J. Taylor, Kristie L. Rose, Peter C. Gareiss, Roberto A. Crespo, Jason Phan, Alex G. Waterson, Stephen W. Fesik

**Affiliations:** ^1^ Department of Biochemistry Vanderbilt University School of Medicine Nashville Tennessee USA; ^2^ Arvinas, Inc. New Haven Connecticut USA; ^3^ Department of Pharmacology Vanderbilt University School of Medicine Nashville Tennessee USA; ^4^ Department of Chemistry Vanderbilt University Nashville Tennessee USA; ^5^ Present address: Ten63 Therapeutics Durham NC USA

**Keywords:** 1H‐13C‐SOFAST HMQC, construct design, E3 ligase, Fem‐1 homolog B (FEM1B), fragment screen, NMR, X‐ray crystal structure

## Abstract

Targeted protein degradation using PROTACs (PROteolysis TArgeting Chimeras) has emerged as a transformative therapeutic strategy, largely relying on a small number of E3 ubiquitin ligases such as CRBN and VHL. However, resistance, toxicity, and poor oral bioavailability limit the utility of PROTACs and highlight the need to expand the E3 ligase toolbox. Fem‐1 homolog B (FEM1B) is a lesser‐known E3 ligase that offers a promising alternative due to its broad expression and ability to recognize diverse degron motifs. Here, we describe the development of a stable construct of FEM1B, the results of a protein‐observed NMR‐based fragment screen using this construct, and the X‐ray structures of some of the fragment hits when bound to the protein. From these results, new PROTACs utilizing FEM1B as the E3 ligase may be discovered, providing an alternative E3 ligase for targeted protein degradation.

## 1 INTRODUCTION

Targeted protein degradation has been utilized for therapeutic approaches by hijacking the ubiquitin‐proteasome system (UPS) using heterobivalent PROTAC molecules (Kramer & Zhang, 2022; Sincere et al., 2023; Yang, Zhao, et al., 2021). These PROTACs consist of a ligand that binds to a target protein, a ligand that recruits the E3 ubiquitin ligase, and a linker joining the two (Yang, Zhao, et al., 2021). PROTACs can induce the formation of a ternary complex between a target protein, the PROTAC, and the E3 ligase resulting in polyubiquitination of the target protein and subsequent degradation through the UPS (Sincere et al., 2023). Many PROTACs have been developed to degrade over 60 disease‐associated proteins, and, to date, at least 22 PROTACs are under investigation in clinical studies (Wang et al., 2024). Although over 600 E3 ligases are known to be expressed in human cells, few of them are used in PROTAC‐mediated targeted protein degradation. Indeed, PROTACs that recruit cereblon (CRBN) and von Hippel Lindau (VHL) are predominant in PROTAC development, specifically for those currently in clinical trials (Gough et al., 2024; Robbins et al., 2024; Zheng et al., 2024).

Although CRBN and VHL are the two primary E3 ligases that have been employed in the discovery of potent PROTACs, expansion of the E3 ligase toolbox will be beneficial for future PROTAC discovery and development (Hughes et al., 2021; Kofink et al., 2022; Kramer & Zhang, 2022). Pre‐clinical studies using PROTACs that recruit CRBN or VHL have observed resistance by downregulation of the E3 ligase or by mutation of the E3 ligase; however, degradation activity could be rescued by utilizing a PROTAC based on a different E3 ligase (Guo et al., 2023). This suggests that having access to multiple E3 ligase‐based PROTACs could overcome the resistance mechanism in some diseases. In addition, PROTACs designed to utilize VHL include a common hydroxyproline chemical moiety, which restricts design and development of orally available PROTAC treatments, while PROTACs utilizing CRBN have a higher instance of toxicity attributed to CRBN off‐target activity (Kramer & Zhang, 2022; Sincere et al., 2023; Wang et al., 2024). Discovering ligands for E3 ligases beyond CRBN and VHL could allow for more flexibility in the discovery of PROTACs to overcome resistance and other issues (Hughes et al., 2021).

Protein fem‐1 homolog B (FEM1B) is a substrate recognition protein of the Cullin‐RING (CUL2) E3 ligase and plays a critical role in regulating proteins in the cell (Chen et al., 2021, 2024; Hughes et al., 2021; Kramer & Zhang, 2022; Manford et al., 2021; Raiff et al., 2025; Zhao et al., 2021). FEM1B is expressed in most tissues and cells, including endothelial cells, liver cells, and squamous epithelial cells of the esophagus and stomach (Bastian et al., 2021). FEM1B plays a crucial role in balancing the reductive stress response in the cell by degrading key proteins involved in redox sensing and management (Manford et al., 2021). Recently, a number of proteins unrelated to redox management, with different recognition motifs, have been identified and characterized as substrates of FEM1B (Timms et al., 2023).

FEM1B binds multiple degron substrates with distinct recognition sequences, which suggests FEM1B has multiple recognition sites for substrate binding. FNIP1 is one such substrate that contains three conserved cysteines that are selectively reduced when reactive oxygen species are depleted in the cell and is essential in detecting oxidative stress. The reduced cysteines of FNIP1 can coordinate with zinc ions and a cysteine (Cys186) in the binding pocket of FEM1B to form a strong interaction between FEM1B and FNIP1, which leads to the ubiquitination and degradation of FNIP1 (Manford et al., 2021). CDK5R1, SMCR8, and PLD6 are a second set of substrates of FEM1B that are recognized by an arginine near the C‐terminus of the degron. The arginine residue of the degrons interacts with acidic residues Asp82 and Asp131 of FEM1B for recognition (Chen et al., 2021; Raiff et al., 2025; Zhao et al., 2021). In addition to these two degron motifs recognized by FEM1B, a third motif has been identified. Utilizing the arginine binding pocket and a hydrophobic pocket composed of Tyr84, Trp93, and Phe130, FEM1B also recognizes a C‐terminal proline‐containing motif. The long, linear degron CCDC89 and BEX3 are examples of substrates with this motif (Chen et al., 2024).

Nomura et al. screened a library of 566 cysteine‐reactive covalent ligands in a competitive fluorescence polarization assay with recombinant mouse FEM1B and a TAMARA‐FNIP1 degron and observed a chloroacetamide molecule (EN106) with a 50% inhibitory concentration (IC_50_) of 2.2 μM. EN106 was shown to covalently bind to Cys186 based on liquid chromatography–tandem mass spectrometry (Henning et al., 2022). To determine if EN106 could be used as the basis for a FEM1B‐based targeted degrader, several BRD4 degraders with an EN106 warhead were synthesized and evaluated. The most active form had a DC_50_ of 250 nM and 94% maximal BRD4 degradation in HEK293T cells (Henning et al., 2022).

**FIGURE 1:**
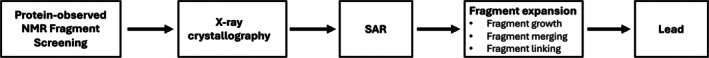
Workflow for fragment‐based drug design. Protein‐observed NMR is used to screen a fragment library and identify low‐affinity binders. X‐ray crystallography defines their binding sites and modes. Combined structural data informs structure–activity relationship (SAR) studies, guiding fragment expansion through growth, merging, or linking. These optimizations can enhance affinity by 100–1000 fold (Harner et al., 2013 ; Yim et al., 2024), advancing compounds toward lead development.

To identify reversible binders to FEM1B, we sought to conduct an NMR‐based fragment screen, using protein‐observed NMR—the method of choice for identifying small molecules that bind to challenging proteins (Figure 1). However, our initial attempts to generate a suitable construct to conduct the screen resulted in unstable proteins. Here we report the preparation of several mutants of FEM1B to identify a suitable protein construct that gave good quality NMR spectra. Due to the size of the protein, we conducted ^1^H‐^13^C HMQC spectra using selective ^13^C‐methyl labeling of Leu, Ile, Val, and Met of FEM1B for the fragment screen. In this screen, several unique fragment hits were identified, and the binding sites were characterized by X‐ray co‐crystal structures. These results offer starting points for the discovery of more potent ligands for FEM1B that may be useful in building FEM1B‐recruiting PROTACs.

## 2 RESULTS

### 2.1 FEM1B construct engineering for NMR‐based fragment screening

The substrate binding domain of FEM1B (residues 1–356, ~42 kDa molecular weight) was prepared for NMR‐based screening. However, this construct (FEM1B WT) was prone to aggregation and was not suitable for the NMR experiments. Consistent with the potential for aggregation, this construct showed a low melting temperature (Tm) of 35.6°C. To reduce the potential for aggregation, we prepared several mutants of FEM1B constructs and evaluated their stability using a thermal shift assay (Table 1).

**TABLE 1:** FEM1B construct characteristics.

Construct	Mutations	pI	Tm ± SD (°C)
FEM1B WT	Wild type	6.5	35.6 ± 0.5
FEM1B‐1	C230S, C244S, C380S	6.3	ND
FEM1B‐2	C94S, C129S, C159S, C230S, C244S, C380S	5.9	ND
FEM1B‐3	L4E, L18E, L281E	6.2	35.0 ± 0.5
FEM1B‐4	L4E, L18E, L281E, R321D	6.1	35.0 ± 0.5
FEM1B‐5	L4E, A21E, L18E, L25S	6.2	38.8 ± 0.3
FEM1B‐6	Y84A, I270N, L281E, R321D	6.2	37.3 ± 0.04
FEM1B‐7	L4E, L18E, L25S, L281E, R321D	5.8	28.7 ± 1.4
FEM1B‐8	L4E, A21E, L25S, I270N, L281E	6.2	34.5 ± 0.2
FEM1B‐9	L4E, A21E, L25S, L281E, R321D	6.1	28.5 ± 1.3
FEM1B‐10	L4E, L18E, L24S, I270N, L281E	6.2	36.3 ± 0.1
FEM1B‐11	L4E, L18E, A21E, L25S, Y84A, I270N, L281E, R321D	5.9	48.6 ± 1.7

*Note*: pI values were calculated for each construct, and the melting temperature (Tm) was determined by thermal shift assays. All data are an average of three replicates, with the standard deviation noted. ND represents no data collected.

Two FEM1B constructs (FEM1B‐1 and FEM1B‐2) were designed to remove free cysteines on the exterior of the protein to prevent disulfide bond formation and prevent aggregation. FEM1B‐3 to FEM1B‐11 were designed to modify hydrophobic residues on the protein surface and reduce the predicted pI away from the pH needed for the experiment. To accomplish this, residues such as Leu, Ile, Val, or Arg were mutated to negatively charged residues (Asp or Glu) to shift the pI to be more acidic. Hydrophobic residues on the surface of the protein were chosen for mutation to also reduce solvent‐exposed hydrophobic patches that may be contributing to the aggregation (Figure S1, Supporting Information). A range of melting temperatures was observed for constructs FEM1B‐3 to FEM1B‐11. The most stable construct was FEM1B‐11, which had the highest melting temperature. This protein was found to be stable at room temperature for over 24 h.

FEM1B‐11 has nine mutations of hydrophobic residues on the protein surface (Figure S1), including Tyr84Ala (Chen et al., 2024). Because Tyr84 plays a role in substrate recognition and could impact fragment binding to FEM1B, construct FEM1B‐12 was prepared with the same mutations as FEM1B‐11 but without the mutation to Tyr84. This construct was also found to be stable and was used to conduct the fragment screen.

### 2.2 Evaluation of FEM1B‐12 binding to known substrate degrons

To ensure the mutations made to FEM1B‐12 did not impact the ability for substrates to bind to the protein, this construct was evaluated for binding to known substrates and compared to wild type FEM1B (Chen et al., 2024). Three known substrates, FNIP, BEX3, and CDK5R, were evaluated for their binding affinities (dissociation constants, K_D_) to FEM1B‐12 using fluorescence polarization (Table S1). FNIP is recognized by a Zn‐binding motif with Cys186 of FEM1B (Manford et al., 2021), CDK5R is an Arg C‐degron recognized by Asp82 and Asp131 (Chen et al., 2021; Chen et al., 2024; Manford et al., 2021), while BEX3 is a C‐terminal proline degron recognized by Tyr84, Trp93, and Phe130 (Chen et al., 2024). These substrate proteins were found to have similar binding affinities to FEM1B‐12 compared to FEM1B WT, suggesting that the binding site of the modified protein is relatively similar to WT FEM1B confirming our selection of this construct to carry out the fragment screen.

### 2.3 Selection and optimization of the NMR method for fragment‐based screening

FEM1B has a molecular weight of ~42 kDa, which is relatively large for protein NMR experiments. Therefore, we attempted to use ^1^H‐^15^N TROSY experiments to conduct the fragment screen (Puthenveetil & Vinogradova, 2019). To evaluate the suitability of the ^1^H‐^15^N‐TROSY experiment, we prepared uniformly ^15^N‐labeled FEM1B‐12. The ^1^H‐^15^N‐TROSY spectrum of 150 μM ^15^N‐labeled FEM1B‐12 was recorded on a 900 MHz NMR (the optimum field strength for TROSY) with a 30‐min acquisition time. The ^1^H‐^15^N‐TROSY spectrum did not show well‐defined resonances even using higher sample concentrations (Figure S2A). Therefore, we resorted to selective ^13^C‐methyl labeling of methyl groups and ^1^H‐^13^C‐methyl SOFAST HMQC experiments. We used 100 μM selective (Leu, Ile, Met, and Val) ^13^C‐methyl labeled FEM1B‐12 and collected the data in 30 min on a 600 MHz NMR (Figure S2B).

### 2.4 Fragment screening with selective ^13^C‐methyl labeled FEM1B

**FIGURE 2:**
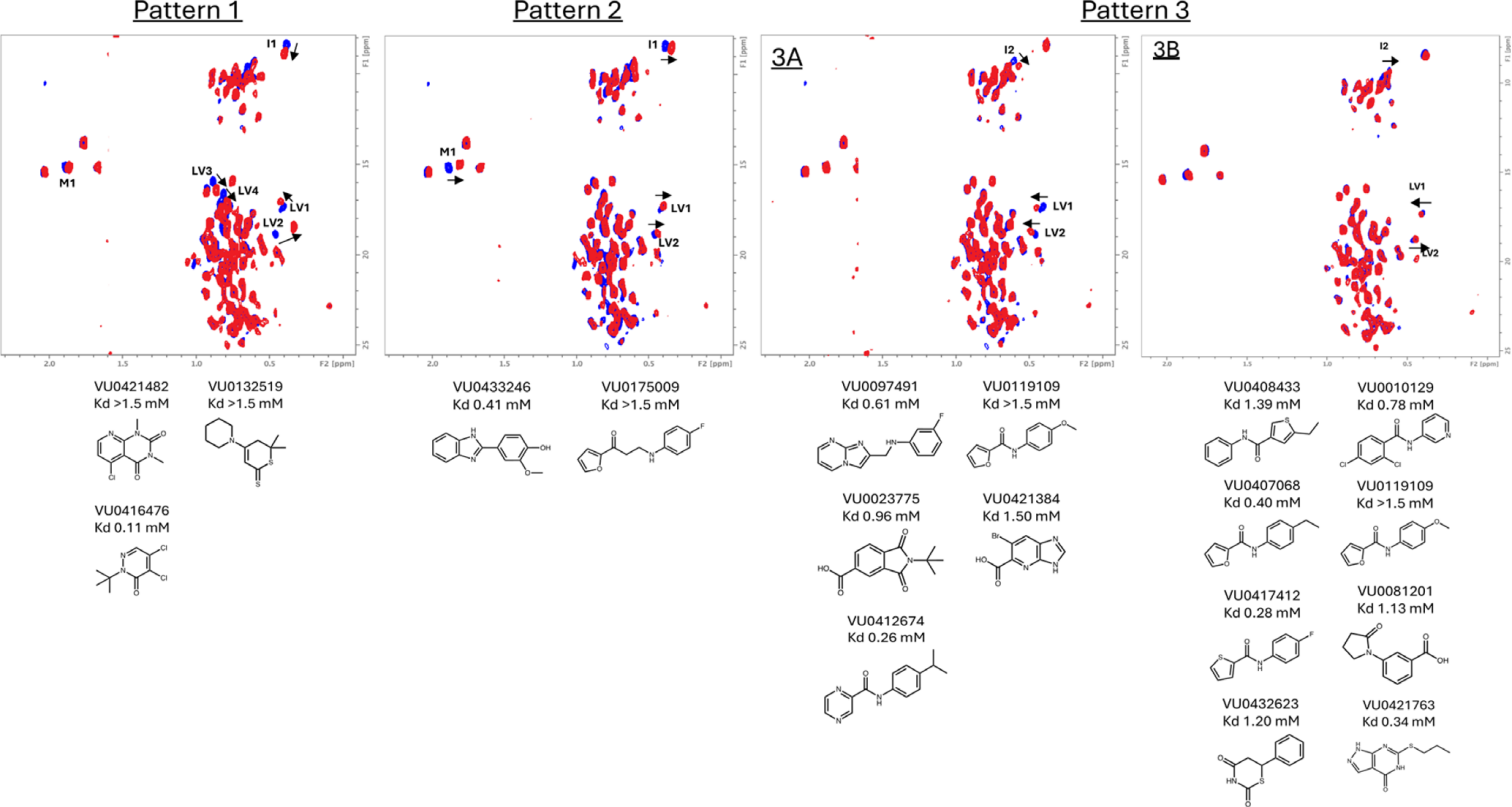
Representative chemical shift patterns for each series of fragments identified to bind to FEM1B‐12 with fragments and dissociation constants that cause each NMR shift pattern represented below the designated shift pattern.

Using ^1^H‐^13^C‐methyl SOFAST HMQC NMR, 4608 structurally diverse fragments were prepared as 12‐fragment mixtures at the concentration of 800 μM per compound and screened against 100 μM FEM1B‐12. The spectra of FEM1B in the presence of the fragment mixtures were compared to the spectrum of ligand‐free FEM1B to identify fragment hits. Twelve‐fragment mixture hits were subsequently deconvoluted by testing individual fragments for binding. In total, 18 fragment hits were identified (3.4% hit rate) in the screen. Three chemical shift patterns were observed among the 18 fragment hits (Figure 2), with pattern three having two sub‐patterns with the same residues shifting in different directions. This suggests multiple binding sites and/or poses for the fragments. The dissociation constants of the fragments were determined by NMR titration using fragment concentrations ranging from 0.0625 to 2 mM (Figure 2) (Henning et al., 2022).

### 2.5 Identification of a covalent fragment that binds to Cys186

**FIGURE 3:**
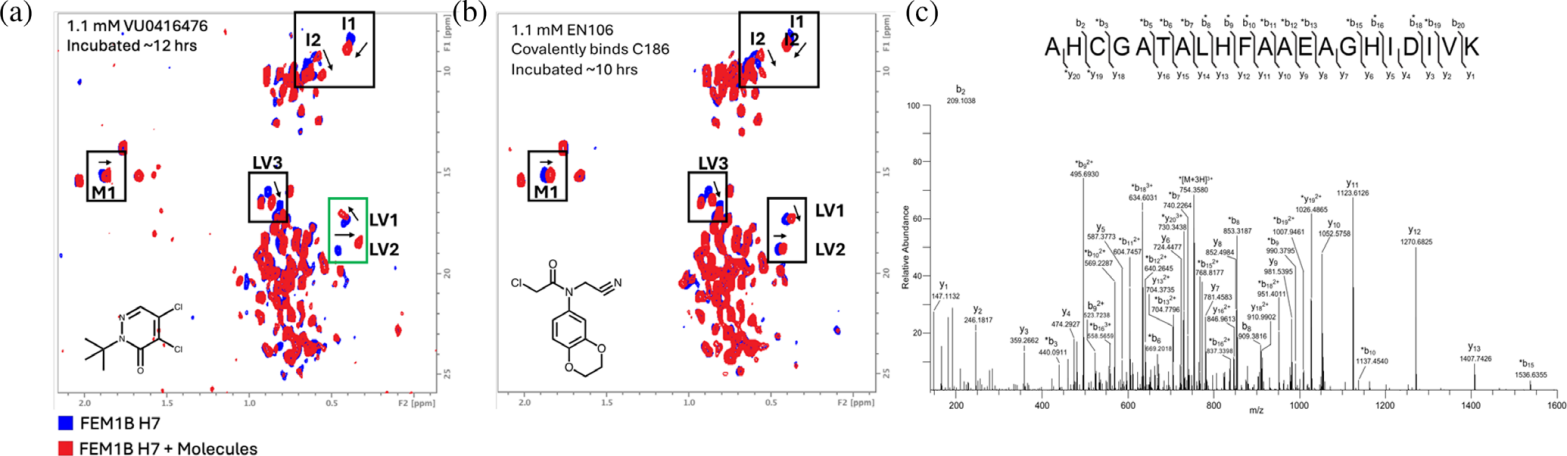
(a) Chemical shift pattern of 100 μM FEM1B‐12 (blue) and 100 μM FEM1B‐12 with 1.1 mM VU0416476 (red) incubated for approximately 12 h. (b) Chemical shift pattern of 100 μM FEM1B H7 (blue) and 100 μM FEM1B H7 with 1.1 mM EN106 (red) incubated for approximately 10 h. The two different chemical shifts are outlined in a green box in the leucine/valine residue shift region. (c) Tandem mass spectrum of VU0416476‐adducted FEM1B‐12 peptide, AHCGATALHFAAEAGHIDIVK. The [M + 3H]^3+^ precursor ion (m/z 772.7085) was selected for fragmentation with higher‐energy collisional dissociation (HCD). The observed b‐ and y‐type product ions are assigned to their corresponding peaks in the mass spectrum. The amino acid sequence is displayed with inter‐residue brackets to indicate fragmentation along the peptide backbone. Asterisks adjacent to product ion assignments denote VU0416476‐modified ions resulting from loss of 56.06 amu upon HCD. Asterisks above labels on the peptide sequence denote b‐type ions that were observed in multiple forms, including ions modified with intact VU0416476 as well as ions that have undergone loss of 56.06 amu. The site of covalent addition of VU0416476 at Cys186 is denoted by 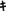.

The chemical shift pattern of FEM1B‐12 in the presence of VU0416476 was similar to the shifts induced by EN106 (Figure 3b), with the direction of the chemical shift perturbation being different for LV1 and in the same direction, but much larger, for LV2 (Figure 3a). Unlike other fragments, VU0416476 showed a slow rate of exchange based on the chemical shift perturbations as a function of molecule concentration (Figure S3) (Bourgeois et al., 2014). This suggested the molecule may bind to FEM1B covalently (Henning et al., 2022).

To assess the potential for covalent binding of VU0416476 to FEM1B, we used LC‐coupled tandem mass spectrometry to identify the site of covalent attachment. After FEM1B was incubated with five times the concentration of VU0416476 for 48 h, the protein was digested with sequencing‐grade trypsin, and peptides were analyzed by LC‐MS/MS. The MS/MS spectrum of the modified peptide (residues 184–204) confirms that VU0416476 covalently binds to Cys186 on FEM1B (Figure 3c).

### 2.6 X‐ray structure of covalent fragment VU0416476 bound to FEM1B

**FIGURE 4:**
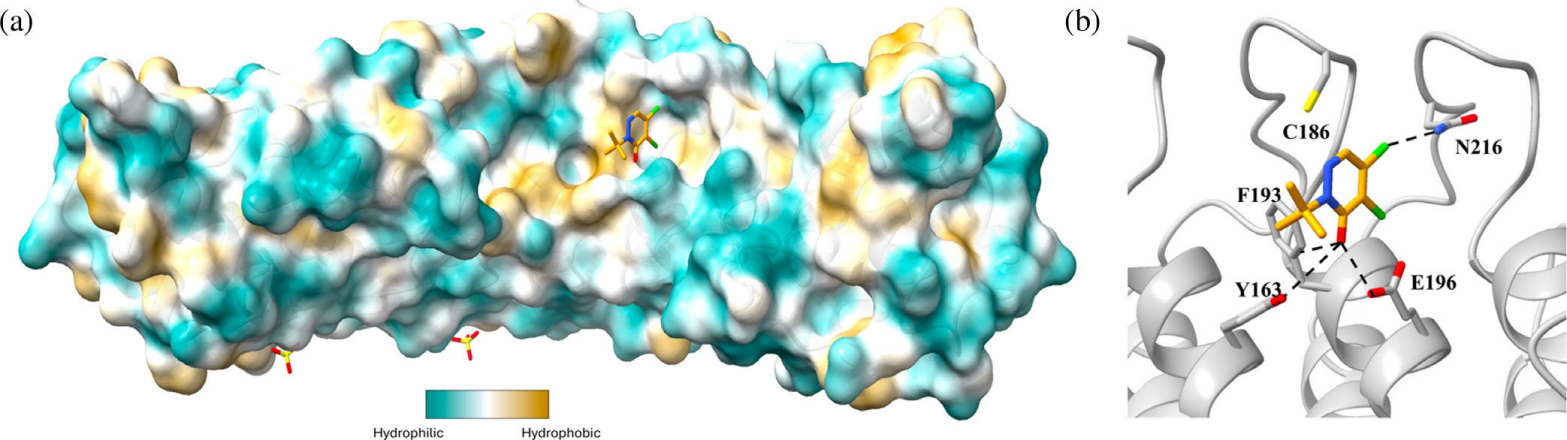
(a) Crystal structure of FEM1B WT:VU0416476 (orange sticks) (PDB ID 9PXP) with surface colored by hydrophobicity. (b) VU0416476 bound to FEM1B WT with nearby residues forming polar contacts represented as sticks.

A co‐crystal structure of FEM1B WT in complex with VU0416476 was solved (Figure 4). In the structure, VU0416476 sits less than 4 Å from Cys186, with the electrophilic carbon of VU0416476 in the correct position for nucleophilic attack by Cys186. Though a covalent bond is not observed in the crystal structure, the conformation of VU0416476, together with the mass spectrometry data, supports this fragment as a covalent binder to FEM1B.

### 2.7 Identification of reversible fragments that bind to FEM1B

Fifteen reversibly binding fragments were identified, with two different chemical shift patterns distinct from the covalent pattern observed with VU0416476 or EN106 bound to FEM1B‐12. Two fragments, VU0433246 and VU0175009, caused chemical shift perturbations characterized as pattern 2. This pattern included the shift of peaks M1 and I1 unique to this binding mode (Figure 2). The other 13 reversible fragments were characterized as chemical shift pattern 3 with two sub‐patterns (Figure 2). The sub‐patterns were differentiated by a difference in the direction of the I2, LV1 and LV2 peak shifts, suggesting a difference in chemical environment upon fragment binding. X‐ray crystallography was used to characterize the binding modes of a subset of these fragments for structure–activity relationship (SAR) analyses.

### 2.8 X‐ray structures of non‐covalent fragment hits bound to FEM1B

**FIGURE 5:**
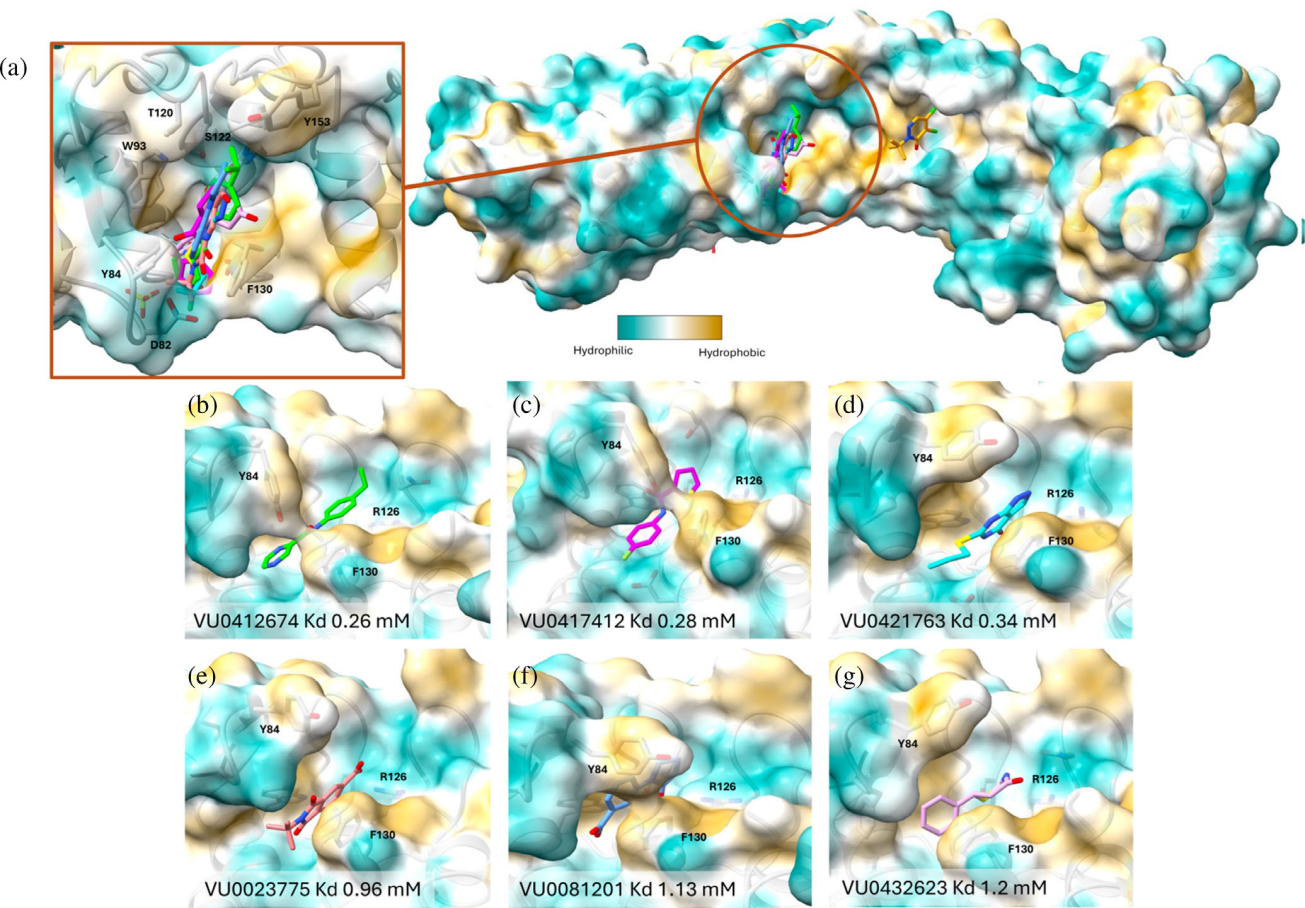
(a) X‐ray crystal structures of all FEM1B:fragment complexes overlaid showing different binding positions of the covalent molecule VU0416476 (orange sticks) (PDB ID 9PXP) and the other six molecules. Surface area is colored by hydrophobicity. (b) Binding pose for VU0412674 (green sticks) (PDB ID 9PQE), (c) VU0417412 (magenta sticks) (PDB ID 9PW8), (d) VU0421763 (cyan sticks) (PDB ID 9PQ9), (e) VU0023775 (coral sticks) (PDB ID 9PXO), (f) VU0081201 (light blue sticks) (PDB ID 9PWJ), and (g) VU0432623 (plum sticks) (PDB ID 9PQA) that bind to the proline‐recognition site of the degron binding pocket.

The X‐ray structures were determined for six additional molecules (VU0412674, VU0417412, VU0421763, VU0023775, VU0081201, and VU0432623) in complex with FEM1B. The six additional molecules which are classified as chemical shift pattern 3 sit in the same binding pocket (Figure 5b–g and S4). An overlay of the X‐ray structures shows the differences in positions for the fragments and the conformational changes of the nearby residues (Figure 5a). Tyr84 is known to be important for the identification of substrate degron targets that include a C‐terminal proline for C‐degron recognition (Sincere et al., 2023). When the most potent non‐covalent molecules, VU0412674 (K_D_ = 0.26 mM) and VU0417412 (K_D_ = 0.28 mM), are bound to FEM1B, Tyr84 forms a polar contact with the amide carbonyl of the molecules. This, along with Phe130, causes this portion of the binding pocket to “close,” locking the fragments in place (Figure 5b,c). VU0421763 (K_D_ = 0.34 mM) (Figure 5d) does not sit in the same closed position, but Phe130 forms a hydrophobic interaction with the pyrazolo pyrimidine ring of the molecule, while Tyr84 sits in a flipped orientation compared to its position with VU0412674 or VU0417412 present. With VU0081201 (K_D_ = 1.13 mM) bound, Tyr84 and Phe130 form the “closed” conformation, but the molecule sits deeper in the substrate pocket and is not locked into position by the conformational changes (Figure 5f). Compounds VU0023775 (K_D_ = 0.96 mM) and VU0432623 (K_D_ = 1.20 mM) both form polar contacts with Arg126 and a hydrophobic interaction with Phe130, but Tyr84 does not form the “closed” position to lock the molecules into the binding pocket.

### 2.9 SAR evaluation of fragment hits and screening of analogs

An analysis of the fragment bound structures of FEM1B provided several hypotheses for changes to the compounds that could improve affinity to the protein. The position of Y84 appears to have a significant impact, with the “closed” position providing an increase in binding affinity by locking the molecule in place. In addition, Arg126 makes polar contacts with some fragments without Y84 in the “closed” position, contributing to the affinity of the fragment. A molecule that exploits both of these characteristics could be capable of tighter binding to FEM1B and thus be utilized as the basis for a PROTAC.

**FIGURE 6:**
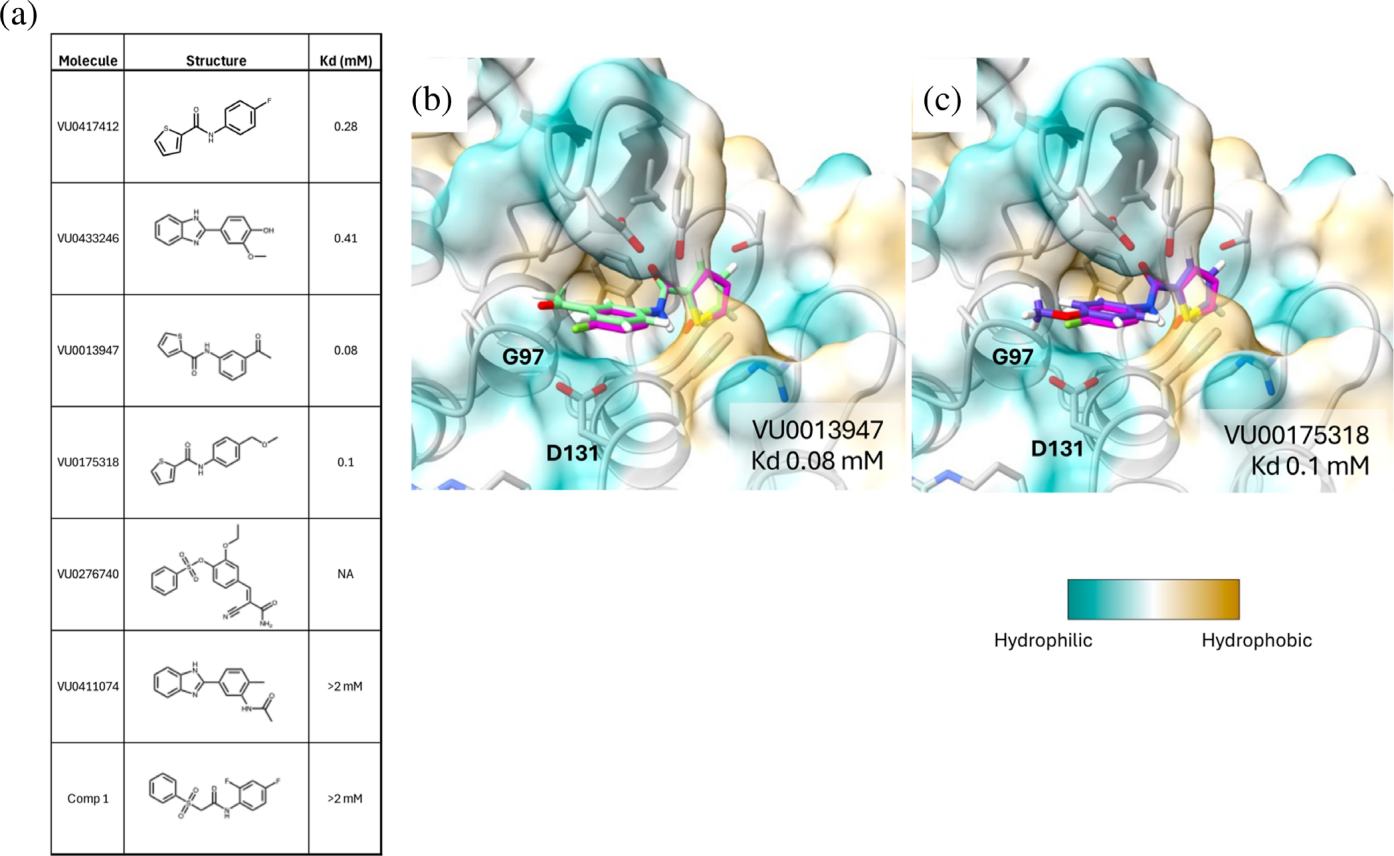
(a) Dissociation constants were determined by NMR titration as previously described. The K_D_ for VU0276740, represented as NA could not be determined due to a low starting concentration that did not achieve saturation. Overlays of docked VU0013947 (light green sticks) (b) or docked VU00175318 (purple sticks) (c) with FEM1B:VU0417412 (magenta sticks). Surface area is colored by hydrophobicity.

We obtained analogs of VU0417412 that may interact with Arg126 or induce the conformational change to generate the “closed” pocket. Sixteen molecules were identified and screened using ^1^H‐^13^C‐methyl SOFAST NMR to ascertain additional binders by chemical shift analysis. Of the 16 molecules screened, five were found to bind to FEM1B. Molecules VU0013947 (K_D_ = 0.08 mM) and VU0175318 (K_D_ = 0.1 mM) were selected based on similarity to VU0417412 and showed higher affinity than the reference molecule by 3.5× and 2.8×, respectively (Figure 6a). These compounds were docked into the protein to evaluate their predicted binding modes. An overlay of the docked molecules with the crystal structure of FEM1B in complex with VU0417412 suggests the carbonyl of the amide on VU0013947 and the ether of VU0175318 could make a polar contact with the backbone of Gly97 and/or the side chain of Asp131 (Figure 6b,c), which may contribute to the increase in affinity.

We also noted that VU0432623 which non‐covalently binds to FEM1B was found to be 8.7 Å from the covalent molecule VU0416476 (Figure S4A). The region between the two fragments is part of the larger substrate binding pocket (Figure S4B), which could allow for the design of a molecule that could link a non‐covalent fragment to the covalent fragment to obtain more specificity.

## 3 DISCUSSION

FEM1B is a CUL2 E3‐ligase that has been shown to be useful for targeted protein degradation using a covalently bound PROTAC (Henning et al., 2022). Based on the limitations of using VHL or CRBN E3‐ligases, the development of PROTACs that utilize other E3‐ligases, like FEM1B, could aid in the design of alternative PROTACs. To search for new chemotypes that bind to FEM1B, protein‐observed NMR‐based fragment screening against selectively ^13^C‐methyl labeled FEM1B was used to identify binders to the protein and determine the dissociation constants for those fragments. Co‐crystal structures of seven fragments in complex with FEM1B were solved, providing insight into the binding modes of these fragments. One molecule, VU0416476, was identified as a covalent binder to an active cysteine in the substrate pocket, binding at the same position as EN106, a previously identified covalent binding ligand that was used to develop the first PROTAC using FEM1B as the E3‐ligase (Henning et al., 2022). The other six fragments bound to the same site in the FEM1B substrate binding pocket, a region typically important for identifying C‐terminal prolines of degron substrates in a non‐covalent manner. These compounds represent the first reported non‐covalent ligands for FEM1B and could be used as starting templates for discovering higher affinity FEM1B ligands for use in PROTACs.

Many examples of the development of small molecule binders with low binding affinity into high affinity molecules can be found in the literature. Diehl and Ciulli reported the K_D_ of a hydroxyproline fragment for VHL to be 10 mM and, upon expansion of the fragment, developed a molecule with K_D_ of 0.09 μM, which was developed into a PROTAC with potent degradation capability (Diehl & Ciulli, 2022). In another example, a fragment was found to bind to E3 ligase suppressor of cytokine signaling (SOCS) with a K_D_ of 0.19 mM and with optimization through SAR was developed into a molecule with KD of 0.5 μM (Yim et al., 2024). These examples represent how fragments with low binding affinity can be developed into potent molecules for PROTAC development.

For FEM1B, examination of the X‐ray structures reveals opportunities for merging and growing the fragments in search of higher FEM1B affinity as well as a potential avenue for linking a non‐covalent fragment to the covalent fragment, which could generate a molecule with better FEM1B specificity and affinity due to having multiple recognition sites for the activator. Together, these strategies may allow for the discovery of a potent FEM1B ligand for use in PROTACs that recruit the ligase to a target protein to accomplish its degradation.

## 4 MATERIALS AND METHODS

### 4.1 Protein expression and purification

All gene synthesis, cloning, and sequencing in this work were conducted by GenScript. FEM1B constructs were cloned into pET28a(+) plasmids. Plasmids were transformed into *E. coli*. BL21(DE3) Rosetta 2 cells and grown on selective agar media supplemented with kanamycin and chloramphenicol. Bacteria were cultured at 37°C in LB broth or M15 minimal media supplemented with ^13^C‐labeled methionine, ^13^C‐α‐ketoisovalerate, and ^13^C‐α‐ketobuterate for regular and isotope‐labeled protein, respectively, until optical density at 600 nm reached 0.6. All FEM1B expression was induced by addition of 1 mM isopropyl β‐d‐1‐thiogalactopyranoside followed by further incubation at 37°C overnight. Cell pellet was harvested by centrifugation at 6000*g* for 30 min and resuspended in lysis buffer (50 mM Tris pH 8.0, 250 mM NaCl, 5 mM ß‐mercaptoethanol) and lysed using a APV2000 Lab homogenizer (SPX Flow) at 600 bar. Cell lysate was centrifuged at 12,000*g* at 4°C for 30 min to remove cell debris. Supernatant was filtered and loaded onto a HisTrap NiNTA column (Cytiva) pre‐equilibrated with lysis buffer. The column was washed with 10× column volume of lysis buffer and then eluted with 500 mM imidazole in the same buffer using a linear gradient from 0% to 100% over 10x column volume. The FEM1B construct was concentrated and loaded to HiLoad 26/600 Superdex75 pg. (Cytiva) and eluted with 20 mM Tris pH 8.0, 150 mM NaCl, 5% glycerol and 1 mM TCEP for crystallography 50 mM sodium phosphate pH 7.5, 50 mM NaCl, 2 mM DTT in deuterated water for NMR experiments. FEM1B concentrations were determined by Pierce 660 nm assay reagent (Thermofisher) using standard bovine albumin serum for making a standard curve (Thermofisher).

### 4.2 Fluorescence polarization method

Fluorescent peptides were synthesized by Genscript: FNIP (Cy5‐RNKSSLLFKESEETRTPNCNCKYCSHPVLG), BEX3 (Cy5‐RELQLRNCLRILMGELSNHHDHHDEFCLMP), and CDK5R (Cy5‐SGSGSGYKKRLLLGLDR). Assays were performed in Corning 3575 black 384‐well plates by mixing 10 μL of FEM1B constructs at varying concentrations, and 10 μL of Cy5‐labeled peptides at a final concentration of 10 nM, in a buffer consisting of 50 mM HEPES pH 7.3, 150 mM NaCl, 0.01% Tween20, and 0.1 mM TCEP. Fluorescence polarization (Ex620 nm, pEm688 nm, sEm688 nm) was measured on an Envision (Perkin Elmer) plate reader. All assays were performed in quadruplicate. Data were analyzed in GraphPad Prism and Kd values were determined using the one site‐total binding model.

### 4.3 NMR experiments

All ^15^N‐TROSY NMR experiments were carried out at 310 K using a 900 MHz Bruker Avance III spectrometer equipped with a TXI/TCI CryoProbe and a Bruker SampleJet. All 1H‐13C‐SOFAST HMQC NMR experiments were carried out at 310 K using a 600 MHz Bruker AV‐III‐600 Spectrometer with a TCI CryoProbe and a Bruker SampleJet. Two‐dimensional ^1^H‐^15^ TROSY spectra of 150 μM FEM1B‐12 and ^1^H‐^13^C‐SOFAST HMQC spectra of 100 μM FEM1B‐12 were recorded using 30‐min acquisition times and analyzed using Topspin 4.1.4 (Bruker).

About 4603 compounds from our in‐house fragment library were screened as mixtures of 12 fragments prepared in 12 96‐well plates. Each NMR sample in 3 mm‐diameter NMR tubes (Bruker) was made of 100 μM of ^13^C‐methyl labeled FEM1B‐12, 800 μM of each fragment, and 5% DMSO‐d_6_ for spectrometer locking. Hit mixtures were identified by comparing the chemical shifts of the backbone resonances to a ligand‐free FEM1B‐12 spectrum and then deconvoluted by screening individual fragments.

HMQC titration experiments were used to determine binding affinity of the fragment hits identified from the screen. The changes in ^1^H‐^13^C chemical shifts of selectively labeled sidechain resonances upon the addition of increasing concentrations of the fragments (0.0625–2 mM) were analyzed. The binding affinities (K_D_s) of the fragments were calculated using the Hill's equation model in Prism 10 (GraphPad).

### 4.4 Crystallography

FEM1B WT was concentrated to 10 mg/mL in crystallization buffer containing 20 mM Tris pH 8.0, 150 mM NaCl, 5% glycerol and 1 mM TCEP. FEM1B WT crystals were formed by hanging drop vapor diffusion against reservoir containing 0.1 M HEPES pH 7.5, 0.25% PEG 8000, 1.2–1.6 M ammonium sulfate, and 0.05–0.1 M sodium thiocyanate. FEM1B WT was incubated with 5 mM fragment for 2 h prior to co‐crystallization. Crystals were formed in drops with 1:1 or 2:1 ratio of protein: reservoir and were cryo‐protected in 20% glycerol in reservoir solution with 5 mM fragment present. X‐ray diffraction experiments were performed at 100 K on beamline 8.2.2 at the Advanced Light Source (ALS), Lawrence Berkely National Laboratory, California, USA. Diffraction data were processed using XDS (Kabsch, 2010). Phasing was accomplished by molecular replacement with Phaser (McCoy et al., 2007) using the structure of apo FEM1B (PDB:7CNG; Chen et al., 2021) as the starting model. Ligand models were built by AceDRG (Long et al., 2017) and manually added to the corresponding electron density. VCB co‐crystal structures were determined by several cycles of refinement using Phenix (Adams et al., 2010) and manual modeling with COOT (Emsley & Cowtan, 2004). Structure figures were prepared with ChimeraX (Meng et al., 2023).

### 4.5 Liquid‐chromatography mass spectrometry experiments

FEM1B incubated with five times the concentration of VU0416476 were brought to a final concentration of 5% SDS, reduced with 20 mM DTT at 55°C for 20 min, and alkylated with 40 mM iodoacetamide for 30 min in the dark at room temperature. The samples were then prepared for digestion on S‐Trap™ micro spin columns (ProtiFi) following the manufacturer's instructions. Aqueous phosphoric acid was added to a final concentration of 2.5%, followed by the addition of 90% methanol containing 100 mM TEAB (S‐Trap binding/wash buffer) at 6× the volume. Samples were loaded onto S‐Trap columns and centrifuged at 4000*g*. Columns were washed four times with binding/wash buffer. FEM1B was digested with 1 μg trypsin (Promega) in 50 mM TEAB, pH 8.0, for 1 h at 47°C. Peptides were recovered from S‐Traps by sequential elution with 40 μL each of 50 mM TEAB, 0.2% formic acid, and 0.2% formic acid in 50% acetonitrile. Eluted peptides were dried in a speed‐vac concentrator, reconstituted in aqueous 0.2% formic acid, and analyzed by LC‐coupled tandem mass spectrometry (LC–MS/MS) using similar methods to those described in the work of Howard et al. (2024). Peptides were loaded onto a C18 reverse phase analytical column using a Dionex Ultimate 3000 nanoLC and autosampler. Mobile phase solvents consisted of 0.1% formic acid, 99.9% water (solvent A) and 0.1% formic acid, 99.9% acetonitrile (solvent B). Peptides were eluted at a flow rate of 350 nL/min, using a 90‐min gradient, and were analyzed on an Orbitrap Exploris 480 mass spectrometer (Thermo Scientific). The data acquisition method consisted of data‐dependent MS/MS and targeted MS/MS for Cys186‐containing peptide precursor ions modified by the addition of 184.0403 amu, corresponding to covalent adduction of VU0416476. MS1 spectra were acquired using an AGC target of 3e6, which was followed by up to 15 data‐dependent MS/MS scans and targeted scan events with AGC targets of 1e5 and 2e5, respectively. HCD collision energy was set to 30 nce. Full scan MS1 and MS/MS spectra of VU0416476‐modified peptide, AHCGATALHFAAEAGHIDIVK, were examined by manual interrogation in Xcalibur Qual Browser software (Thermo Scientific). Identifications of [M + 3H]^3+^ and [M + 4H]^4+^ peptide ions of VU0416476‐adducted AHCGATALHFAAEAGHIDIVK were confirmed, including the localization of VU0416476 at Cys186.

### 4.6 Ligand docking

Ligand docking was performed using Schrodinger Maestro (Schrödinger Release 2025‐2: Glide, Schrödinger, LLC, New York, NY, 2025.) while docking into X‐ray crystal structure FEM1B in complex with VU0417412 (PDB ID 9PW8) (Friesner et al., 2004, 2006; Halgren et al., 2004; Yang, Yao, et al., 2021).

## AUTHOR CONTRIBUTIONS

**Jade M. Katinas:** Investigation; writing – original draft; data curation; formal analysis. **Kangsa Amporndanai:** Writing – review and editing; formal analysis; methodology. **Ashley J. Taylor:** Methodology; data curation. **Kristie L. Rose:** Writing – original draft; validation; formal analysis; data curation. **Peter C. Gareiss:** Data curation; writing – original draft; methodology; formal analysis; validation. **Roberto A. Crespo:** Data curation; formal analysis; writing – review and editing; validation. **Jason Phan:** Investigation; methodology; supervision. **Alex G. Waterson:** Supervision; conceptualization; funding acquisition; writing – review and editing; project administration; resources. **Stephen W. Fesik:** Project administration; supervision; resources; formal analysis; writing – review and editing; funding acquisition; investigation; conceptualization.

## CONFLICT OF INTEREST STATEMENT

The authors declare no competing financial interest.

## Supporting information


**Data S1:** Supporting information.

## Data Availability

The data that support the findings of this study are available from the corresponding author upon reasonable request.
