# Supplementary material for: Nuclear Magnetic Resonance‐based fragment screen of the E3 ligase Fem‐1 homolog B
Source: Protein Sci. 2025 Nov 13;34(12):e70365. doi: 10.1002/pro.70365 (PMC12612594; doi:10.1002/pro.70365)
Supplement: Supplementary file 1 — Data S1: Supporting information. [file PRO-34-e70365-s001.docx]

**NMR-based Fragment Screen of the E3 Ligase Fem-1 homolog B (FEM1B)**

*Jade M. Katinas^1^, Kangsa Amporndanai^1^, Ashley J. Taylor^1^, Kristie L. Rose^1^, Peter C. Gareiss^4^, Roberto A. Crespo^4,#^, Jason Phan^1^, Alex G. Waterson^2,3^, Stephen W. Fesik ^1,2,3*^*

^1^ Department of Biochemistry, Vanderbilt University School of Medicine, Nashville, Tennessee, 37232-0146, United States.

^2^ Department of Pharmacology, Vanderbilt University School of Medicine, Nashville, Tennessee, 37232-6600, United States.

^3^ Department of Chemistry, Vanderbilt University, Nashville, Tennessee, 37235, United States.

^4^ Arvinas, Inc., New Haven, CT, 06511, United States.

^#^ Current Association: Ten63 Therapeutics, Durham, NC, 27703, United States.

* Corresponding author

**Corresponding Author Contact**

Stephen W. Fesik
Vanderbilt University School of Medicine, Department of Biochemistry
2215 Garland Avenue, 607 Light Hall, Nashville, Tennessee, 37232-0146;
Orcid.org/0000-0001-5957-6192; Phone: +1 (615) 322-6303; Fax: +1 (615) 875-3236;
Email: [Stephen.fesik@vanderbilt.edu](mailto:Stephen.fesik@vanderbilt.edu)

**Running title: NMR-based fragment screen of the E3 ligase FEM1B**

**Manuscript pages: 20**

**Supplementary material pages: 6**

**Tables: 1**

**Figures: 5**

**Supplementary Material –**

**X-ray data collection and refinement statistics for FEM1B bound to fragments**

**Compound** **VU0416476 VU0412674 VU0417412 VU0421763**

**PDB ID 9PXP 9PQE 9PW8 9PQ9**

**Data Collection**

Space Group P4 2_1_ 2 P4 2_1_ 2 P4 2_1_ 2 P4 2_1_ 2

a, b, c (Å) 129.54, 129.54, 140.19 130.01, 130.01, 140.43 123.71, 123.71, 137.79 124.43, 124.43, 138.64

⍺, β, 𝛄 (°) 90, 90, 90 90, 90, 90 90, 90, 90 90, 90, 90

Resolution (Å) 47.57-3.00 47.40-3.10 46.03-2.80 46.30-2.93

(3.08-3.00) (3.24-3.10) (2.85-2.80) (3.06-2.93)

R_merge_ (%) 0.236 (4.54) 0.236 (3.175) 0.413 (3.976) 0.576 (4.258)

Mean I/𝜎I 14.6 (0.6) 16.1 (0.9) 11.6 (1.0) 8.2 (0.9)

Completeness (%) 97.63 (96.91) 100 (100) 99.7 (100) 100 (100)

Redundancy 13.0 (12.3) 13.1 (13.4) 26.0 (24.7) 25.7 (24.5)

**Structure Refinement**

No. Reflections 24,143 (1702) 22,302 (2731) 26,922 (1478) 23,761 (2943)

R_work_/R_free_ 1.01 (1.08) 0.826 (0.913) 0.886 (0.974) 0.852 (0.960)

R.m.s. deviations

Bond length (%) 0.004 0.005 0.007 0.004

Bond angles (%) 0.949 1.04 1.21 0.802

Ramachandran

Favored (%) 89.38 83.51 90.44 92.16

Allowed (%) 7.08 13.62 7.44 7.39

Outliers (%) 3.54 2.87 2.12 0.45

**Compound** **VU0023775 VU0081201 VU0432623**

**PDB ID 9PXO 9PWJ 9PQA**

**Data Collection**

Space Group P4 2_1_ 2 P4 2_1_ 2 P4 2_1_ 2

a, b, c (Å) 129.84, 129.84, 139.97 128.54, 128.54, 139.75 129.00, 129.00, 139.77

⍺, β, 𝛄 (°) 90, 90, 90 90, 90, 90 90, 90, 90

Resolution (Å) 47.60-3.05 47.30-3.00 47.40-2.90

(3.11-3.05) (3.14-3.00) (3.02-2.90)

R_merge_ (%) 0.308 (3.336) 0.454 (3.200) 0.230 (5.120)

Mean I/𝜎I 14.1 (0.9) 9.4 (0.8) 14.4 (0.6)

Completeness (%) 100 (99.9) 99.7 (99.9) 100 (100)

Redundancy 12.8 (12.6) 11.1 (10.6) 12.9 (12.5)

**Structure Refinement**

No. Reflections 23,199 (1331) 23,703 (2932) 26,155 (2792)

R_work_/R_free_ 0.838 (0.944) 0.853 (0.986) 0.826 (0.936)

R.m.s. deviations

Bond length (%) 0.005 0.004 0.005

Bond angles (%) 1.036 0.61 1.001

Ramachandran

Favored (%) 87.95 91.98 88.8

Allowed (%) 9.64 8.47 8.93

Outliers (%) 2.41 1.06 2.27

**Table S1**. Impact of mutations on dissociation constants for stable FEM1B-12. The dissociation constants of FEM1B WT compared to FEM1B-12 with three degrons with different binding modes were evaluated by fluorescence polarization.

**Substrate Kd (nM)**

**FEM1B Construct FNIP BEX3 CDK5R**

FEM1B WT 28 18 143

FEM1B-12 36 26 149


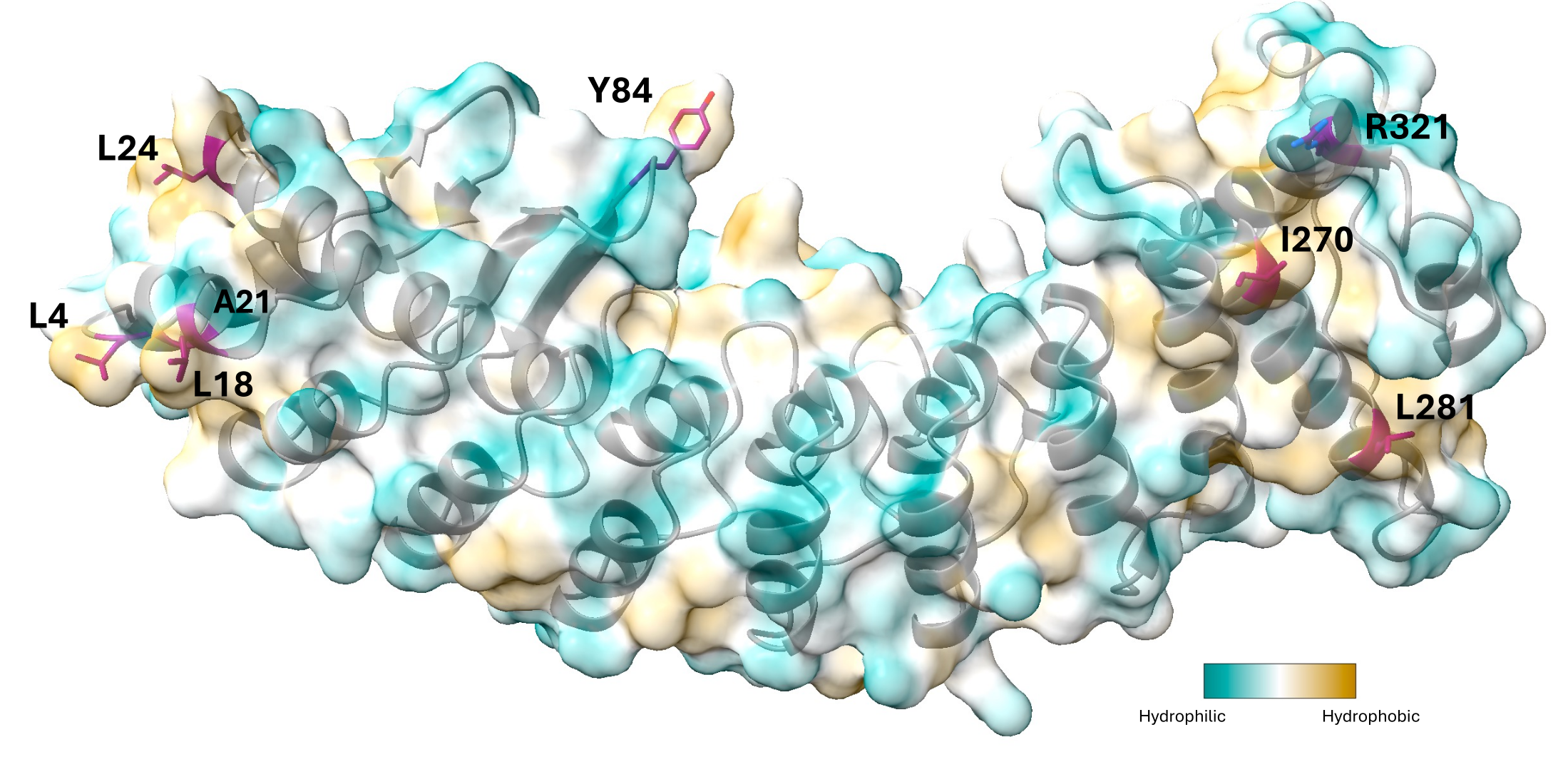


**Figure S1. Location of mutations to FEM1B to generate stable constructs FEM1B-11 and FEM1B-12.** Hydrophobic residues in patches on the exterior of FEM1B were identified and mutated to improve protein stability. Mutated residues are represented as purple sticks, and the protein surface is colored by hydrophobicity.


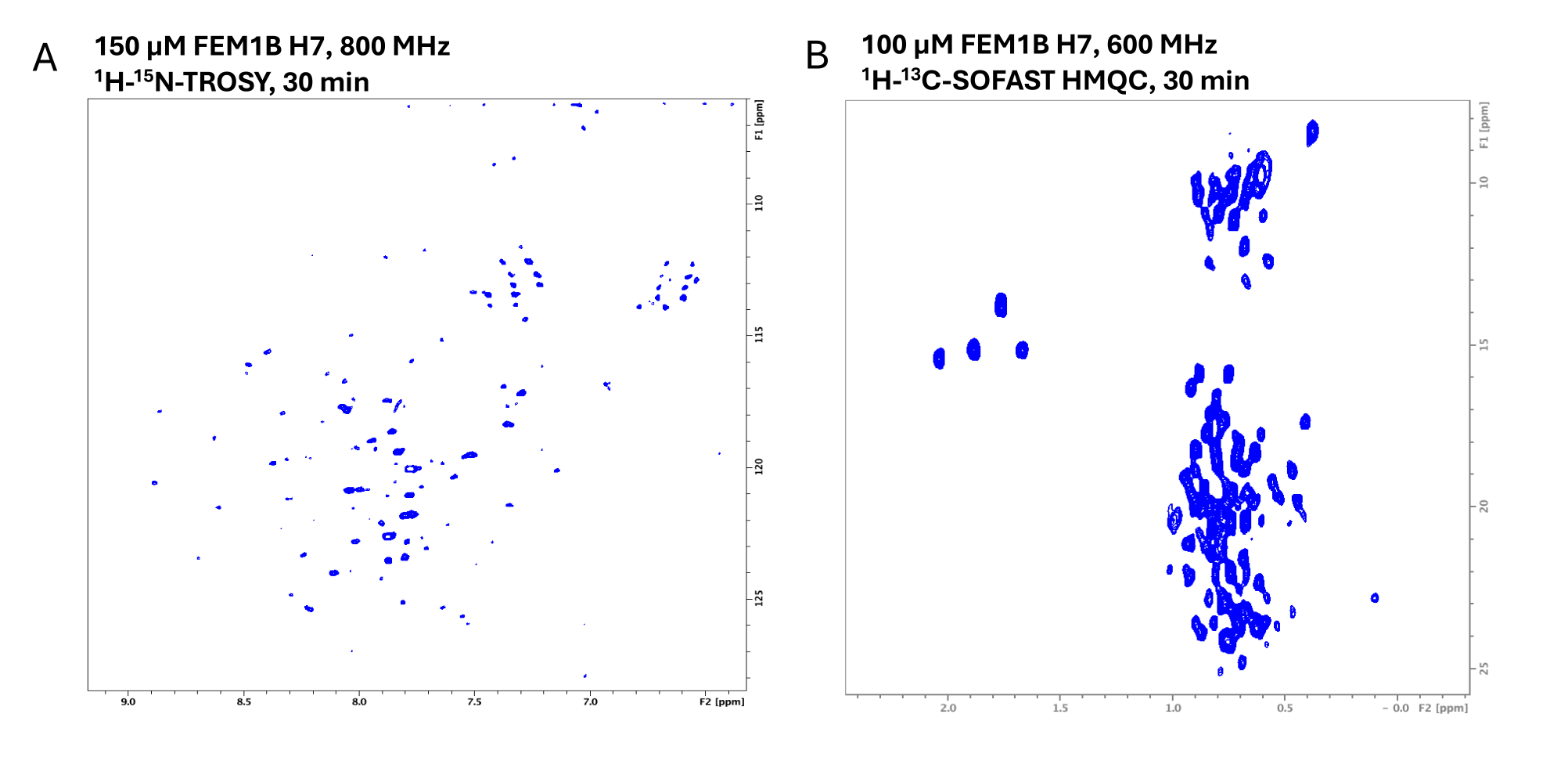


**Figure S2. Comparison of ^1^H-^15^N-TROSY NMR spectra and ^1^H-^13^C-SOFAST HMQC NMR spectra.** (A) ^1^H-^15^N-TROSY spectra collected on the 900 MHz NMR with 150 µM FEM1B-12 and (B) ^1^H-^13^C-SOFAST HMQC spectra collected on the 600 MHz NMR with 100 µM FEM1B-12 with 30-minute acquisitions.


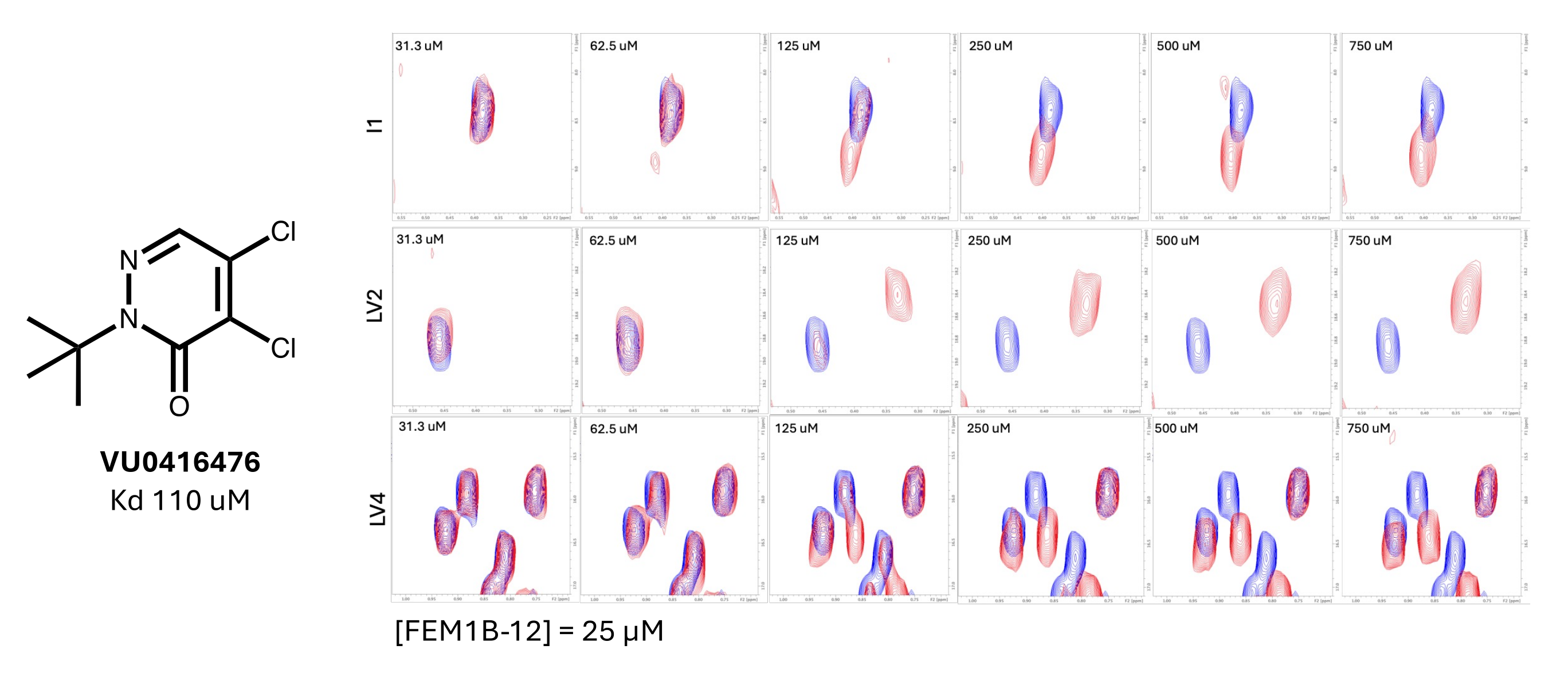


**Figure S3. ^1^H-^13^C-SOFAST HMQC Chemical Perturbations of FEM1B-12 with Addition of VU0416476.** Slow exchange was identified in the chemical shift pattern with the titration of VU0416476, as characterized by the appearance of a new peak caused by chemical shift perturbation upon binding of the fragment and rapid disappearance of the unbound chemical shift with increased fragment concentration.


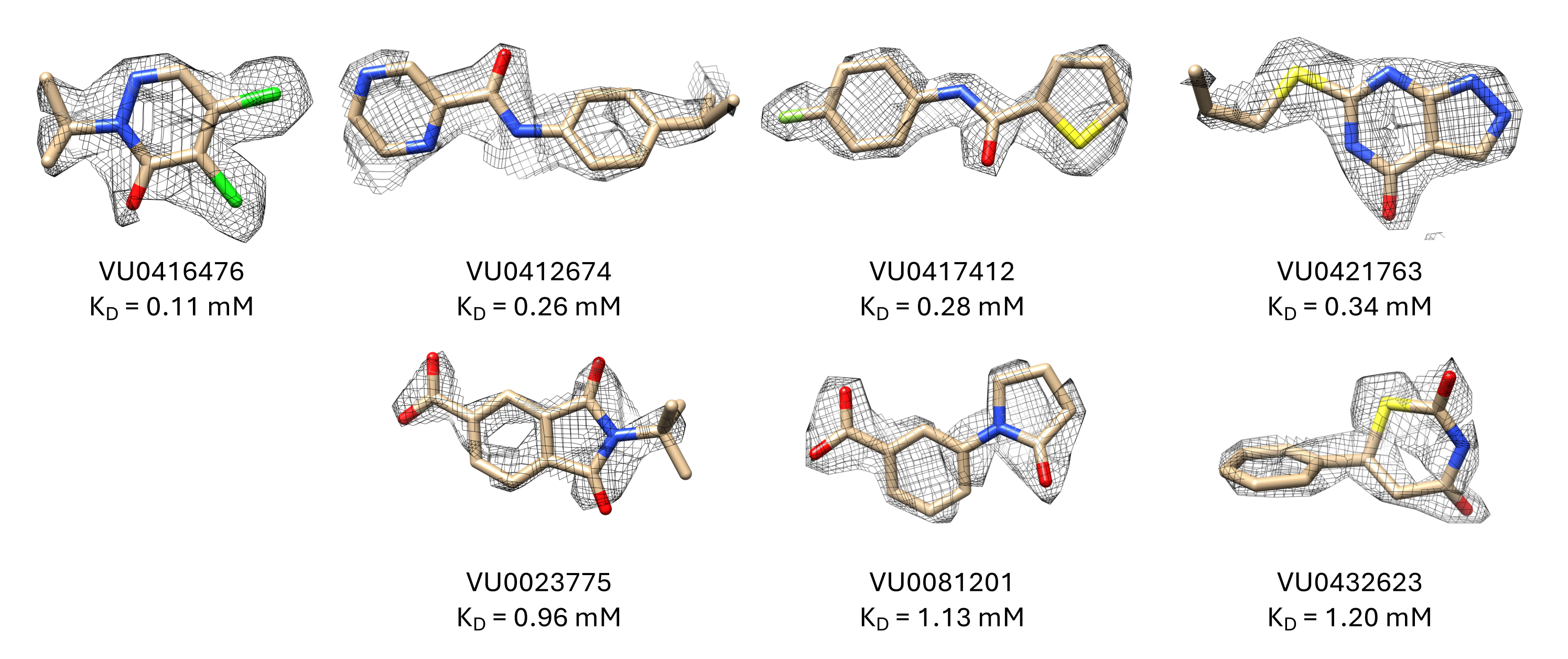


**Figure S4. Electron density of ligand binding poses for fragment hits.** Fragments from structures of FEM1B in complex with fragment hits fit into omit maps with RMSD 1.0.
